# Psychosocial Interventions for People With Dementia and Their Caregivers in Primary Care

**DOI:** 10.1093/geroni/igab046.2968

**Published:** 2021-12-17

**Authors:** Lou Frankenstein, Georg Jahn

**Affiliations:** Chemnitz Technical University, Chemnitz, Sachsen, Germany

## Abstract

Psychosocial interventions, such as occupational and behavioral therapy are effective opportunities to support people with dementia and their caregivers in adapting to the cognitive and behavioral changes and the resulting challenges in everyday life they are facing. However, psychosocial interventions do not seem to have found their way into routine care yet. We wanted to get an insight into the knowledge and attitudes general practitioners have about occupational and behavioral therapy. In an online survey we asked medical students about the relevance of dementia, occupational therapy, and behavioral therapy during their studies. In another online survey we asked practitioners what they had learned about these topics and to what extent they are making use of psychosocial interventions. Then semi-structured interviews were carried out with general practitioners all over Germany, exploring their experiences with dementia and psychosocial interventions in primary care as well as their expectations regarding interdisciplinary cooperation. It became obvious that psychosocial interventions are not conveyed sufficiently within medical school. A lack of occupational therapy prescriptions for people with dementia seemed to result from uncertainties regarding the content of the approach and the budgeting of the prescriptions. Barriers for prescriptions of behavioral treatment were a lack of therapy places and the perceived inadequacy of the approach for this target group. General retentions to invest in people with dementia were expressed. These obstacles need to be overcome in order to provide optimal care for people with dementia and their family caregivers.

